# Randomised trial of proton vs. carbon ion radiation therapy in patients with low and intermediate grade chondrosarcoma of the skull base, clinical phase III study

**DOI:** 10.1186/1471-2407-10-606

**Published:** 2010-11-05

**Authors:** Anna V Nikoghosyan, Geraldine Rauch, Marc W Münter, Alexandra D Jensen, Stephanie E Combs, Meinhard Kieser, Jürgen Debus

**Affiliations:** 1 Dept. of Clinical Radiology, University of Heidelberg, INF 400, 69120 Heidelberg, Germany; 2 Institute of Medical Biometry and Informatics, University of Heidelberg, INF 305, 69120 Heidelberg, Germany

## Abstract

**Background**

Low and intermediate grade chondrosarcomas are relative rare bone tumours. About 5-12% of all chondrosarcomas are localized in base of skull region. Low grade chondrosarcoma has a low incidence of distant metastasis but is potentially lethal disease. Therefore, local therapy is of crucial importance in the treatment of skull base chondrosarcomas. Surgical resection is the primary treatment standard. Unfortunately the late diagnosis and diagnosis at the extensive stage are common due to the slow and asymptomatic growth of the lesions. Consequently, complete resection is hindered due to close proximity to critical and hence dose limiting organs such as optic nerves, chiasm and brainstem. Adjuvant or additional radiation therapy is very important for the improvement of local control rates in the primary treatment. Proton therapy is the gold standard in the treatment of skull base chondrosarcomas. However, high-LET (linear energy transfer) beams such as carbon ions theoretically offer advantages by enhanced biologic effectiveness in slow-growing tumours.

**Methods/Design**

The study is a prospective randomised active-controlled clinical phase III trial. The trial will be carried out at Heidelberger Ionenstrahl-Therapie (HIT) centre as monocentric trial.

Patients with skull base chondrosarcomas will be randomised to either proton or carbon ion radiation therapy. As a standard, patients will undergo non-invasive, rigid immobilization and target volume definition will be carried out based on CT and MRI data. The biologically isoeffective target dose to the PTV (planning target volume) in carbon ion treatment will be 60 Gy E ± 5% and 70 Gy E ± 5% (standard dose) in proton therapy respectively. The 5 year local-progression free survival (LPFS) rate will be analysed as primary end point. Overall survival, progression free and metastasis free survival, patterns of recurrence, local control rate and morbidity are the secondary end points.

**Discussion**

Up to now it was impossible to compare two different particle therapies, i.e. protons and carbon ions, directly at the same facility in connection with the treatment of low grade skull base chondrosarcomas.

This trial is a phase III study to demonstrate that carbon ion radiotherapy (experimental treatment) is not relevantly inferior and at least as good as proton radiotherapy (standard treatment) with respect to 5 year LPFS in the treatment of chondrosarcomas. Additionally, we expect less toxicity in the carbon ion treatment arm.

**Trial Registration**

ClinicalTrials.gov identifier: NCT01182753

## Background

Low and intermediate grade chondrosarcomas (9-13% of all malignant bone tumours) are relative rare bone tumours. In 5-12% of all cases the chondrosarcomas are localized in head-and-neck region [1]. The typical sites of skull base lesions are temporo-occipital junction, parasellar area, spheno-ethmoid region and clivus [2,3]. Due to hystopathological type chondrosarcomas are divided into Grade 1 to 3 tumours according to mitotic rates (WHO classification) with 3 histological subgroups: classic, mesenchymal and myxoid [2]. The mesenchymal type has more aggressive growth behaviour and is associated with a poorer prognosis. Histological differentiation from chordomas is often difficult and must contain immunhistochemical staining [2,4]. Chordoma is immunopositive for epithelial markers like cytokeratin and endothelial membrane antigen (EMA), whereas chondrosarcoma is negative for both. Both chordomas and chondrosarcomas can be positive for S-100 and vimentin [5].

Most patients diagnosed are over the age of 40 years. Low grade chondrosarcoma has a low incidence of distant metastasis but is potentially lethal disease. Thus, local therapy has a crucial importance in the treatment of skull base chondrosarcomas. Surgical resection is the primary treatment standard. Unfortunately the late diagnosis and diagnosis at the extensive size of the tumours are common due to the slow growth kinetics; most patients are asymptomatic, or develop symptoms at a late stage of the disease. Consequently, complete resection is hindered due to close proximity to critical and hence dose limiting organs for radiation therapy i.e. optic nerves, chiasm and brainstem. Adjuvant or additional radiation therapy is very important for the improvement of local control rates in the primary treatment even after complete resection (no compartment resection possible). Anyhow, chondrosarcoma have a better outcome and prognosis compared to chordoma [6,7].

Chondrosarcomas are commonly radioresistant [8], and high local doses are required for long-term local control. Image guidance in conformal precision radiation therapy provides a safe technique in the treatment of base of skull tumours [9]. The highest dose conformality is possible using particle therapy with heavy ions and protons due to inverted dose profile allowing steep dose gradients and therefore providing further benefit in reducing toxicity and irradiation safety.

It is brightly accepted, that proton therapy can be considered the gold standard for treatment of rare skull-base tumours like chordoma and low grade chondrosarcoma [10]. Loma Linda University Medical Center (LLUMC) [8] the Massachusetts General Hospital (MGH) in Boston [11] have the longest experience in proton therapy for these entities. 3-year local control for chondrosarcomas after fractionated proton radiation therapy in 25 patients at LLUMC was 94% and the actuarial 5 year survival rate was 100% respectively [11].

The outcome in 229 chondrosarcomas treated with a combination of proton and photon therapy at MGH/HCL shows 5-and 10-year local progression free survival rate of 98% and 94% for chondrosarcomas respectively. The results are significantly better than in chordomas treated at the same institution [8].

Proton therapy results from PSI in Villingen, Switzerland were published by Weber et al. and Ares et. al. The data showed 3- and 5-year local control rates of 100% and 94%, respectively. 64 patients, among them 22 patients with chondrosarcoma were treated to a median target dose of 68.4 GyE. The 3-year actuarial overall survival rate for the chondrosarcoma patients was 91% [12,13].

Carbon ions though, have a higher biological effectiveness than either protons or photons, which is important in case of radioresistant tumours [14].

Carbon ion therapy is available at the National Institute of Radiological Sciences (NIRS) in Japan and at Heidelberger Ionenstrahl-Therapie (HIT) centre. The report of our Japanese colleagues experience is limited to 40 patients with chordoma and chondrosarcoma of the skull base. The patients could be treated effectively and without serious side effects [15]. NIRS beam delivery technique relies on passive scanning necessitating various modulators to adjust for treatment depth and tissue inhomogeneities within the beam path.

Our experience is based on the clinical work at the Gesellschaft für Schwerionenforschung (GSI) Darmstadt in Germany up to July 2008. In comparison to the Japanese centres, the facility at GSI as well as the HIT relies on active beam delivery using the raster-scan technique. About 300 patients with base of skull chordomas and chondrosarcomas have been treated so far. Initially, these patients were treated within a clinical Phase I/II study. After the study was able to demonstrate successful treatment, carbon ion therapy became approved as the best therapy available in Germany. The actuarial local control rates for the chondrosarcoma patients after 3 and 5 years was 96.2% and 89.8% respectively, the 5 year overall survival rates was 98.2% [16].

## Methods/Design

The study plan has been submitted to the ethics committee of the Medical faculty Heidelberg and is already approved. Also the positive vote of Bundesamt für Strahlenschutz (the governmental authority for radiation protection in Germany) has been already obtained.

The study is a double arm prospective randomised clinical phase III study of patients with low/intermediate grade chondrosarcomas of the skull base. Study patients are selected according to the inclusion criteria of the study protocol. After careful review of the patient reports and results of the additional examinations eligibility of a patient will be determined.

The randomisation will be done using the on-line randomisation tool (Randomizer.at) which is self-serve and runs exclusively on the Internet. The randomisation will be performed regarding treatment arms A and B. Patients will be randomized to the treatment groups with an equal allocation ratio of 1:1.

As this is an open-label study there will be no blinding of treatment assignment.

### Primary objectives of the study

The primary objective of this study is to evaluate, if the innovative therapy (carbon ion irradiation) in chondrosarcomas is not relevantly inferior to the standard proton treatment with respect to the 5 year LPFS rate defined as time from the randomisation to observed local recurrence. Withdrawals, lost to follow-ups and patients for whom no event has occurred at study termination are treated as censored observations. The censoring date is given by the last known date at which no event has occurred for the respective patient. Local recurrence defined as MRT or CT - morphological tumour progress in the former irradiated region. It is assumed that the LPFS rate for the proton therapy is 90%.

### Secondary objectives of the study

Assessment of overall survival, progression free and metastasis free survival, patterns of recurrence, local control rate and morbidity (acute and late toxicity (Common Terminology Criteria for Adverse Events: CTCAE V4.0, RTOG/EORTC for late effects)) are the second objectives of the study. Plan quality (target coverage, sparing of organs at risk, integral dose) is also a matter of interest.

### Inclusion criteria

- Karnofsky Performance Score ≥60%

- Age >18 years and <80 years

- Informed consent signed by the patient

- Histological confirmation of low/intermediate grade chondrosarcoma with infiltration of the skull base.

### Exclusion criteria

- Inability to understand the aims of the study, no informed consent

- Prior RT of skull base region

- Other malignancies with disease-free interval < 5 years (excepting pre-cancerous lesions)

- Participation in another trial

- Pregnancy

- Simultaneous CHT or Immunotherapy.

### Study concept

Pre-treatment examination such as history and physical examination including neurological status, histological confirmation of chondrosarcoma, reference-histopathology if necessary, ophthalmologic examination by optic nerve, chiasm infiltration or by contiguity, audiometry by auditory channel infiltration or by contiguity, endocrinological examination by contiguity to sella turcica region and MR - Imaging (before) and after operation will be done or/and collected.

Patients with histologically confirmed low and intermediate grade chondrosarcoma and infiltration of the skull base which are willing to participate will be included into our study after verification of the eligibility centrally at the HIT trial center. These patients are subsequently randomized to one of the two treatment arms (arm A: carbon ion therapy, arm B: proton therapy).

Carbon ion therapy (Arm A) will be aplicated with a total target dose of 60 Gy E ± 5% to the PTV1. The PTV2 will receive a total carbon ion dose of 45 Gy E.

The patients entered in Arm B will receive proton therapy with the same target definition concept. The total proton dose will be 70 Gy E ± 5%. The PTV2 will receive a total dose of 50 to 56 Gy E in conventional fractionation.

Accrual period for the trial will be approximately 7 years. Our study design contains one interim analysis after observation period of approximately 5.5 years. The study will be terminated early in case of interim analysis showing 5% smaller rate of the 5 year LPFS of the experimental treatment (carbon ion therapy) in respect to the 5 year LPFS rate of standard treatment (proton therapy). If it is not a case the study ends with the enrollement of planned 154 patients. Definite assessments of 5 year LPFS, primary and secondary endpoints will be performed 12 years after completion of radiation therapy.

### Reference Committee

In order to monitor specific aspects of the current trial the following Reference Data Monitoring Committee (DMC) will be established. The DMC will be composed of independent experts in the field of radiation oncology, assessing the progress of the trial and available safety data. The mission of the DMC will be to ensure the ethical conduct of the trial and to protect the safety interests of patients.

The DMC will meet on a regular basis, i.e. once a year. Based on its review of available safety data (CRFs) the DMC will provide the sponsor with written recommendations regarding trial modification, continuation or termination.

## Treatment planning and radiation therapy

Patients will be immobilized using a precision head mask to ensure high repositioning accuracy of the target volume and adjacent structures for carbon ion and proton RT. The treatment planning CT (obligate native CT, CT with contrast facultative) and MR-Examination (MRT - compulsory sequences - axial T1 post gadolinium and T2 fat saturated or Flair fat saturated) will be performed in treatment position using the immobilisation device and will be co-registered. The treatment planning CT will consist of continuous 3 mm slices obtained in a stereotactic or virtual simulation set-up.

The delineation of organs at risk and target volume definition will be done on the basis of CTs and MRI scans. The CTV1 should include the GTV (entire residual tumour) and a 1-2 mm safety margin. CTV2 includes the CTV1 with individual safety margin based on surgical and histological reports, and MR-images to account for subclinical disease. The PTV will created adding safety margins around the CTV individually for each patient. The following organs at risk will be defined: eyes, optic nerves, chiasm, brainstem, spinal cord obligatory, temporal lobes, mandible, salivary glands and others facultative. An overlap of the CTV and the OAR needs to be avoided.

### Carbon ion/Proton RT

Carbon ion RT planning is performed using the treatment planning software including biological plan optimization for carbon ions. Two to maximum four irradiation fields will be chosen. At HIT the intensity-controlled raster-scan system will be used for beam application. Considering the tolerance dose to organs at risk a dose of 60 Gy E ± 5% in 19-21 fractions for carbon ions and 70 Gy E ± 5% in 34-36 fractions for protons will be prescribed to the maximum of the calculated dose distribution for the target volume (CTV1). The dose prescription used is related to the isoeffective dose Gy E using daily fractions of 3 Gy E and a weekly fractionation of 4-6 × 3 Gy E for carbon ions. For proton therapy daily fractions of 2 Gy E and a weekly fractionation of 4-6 × 2 Gy E will be used. Treatment planning aims at coverage of the CTV1 and CTV2 with the 95%-isodose line of the prescribed dose.

Evaluation of DVH for the dose distribution will be performed with regards to assess plan quality.

Positioning accuracy will be controlled for each fraction using orthogonal x-rays or cone-beam-CTs. Set-up deviations > 2 mm will be corrected prior to irradiation by correction with the vector of the robotic table.

### Dose constraints to organs at risk for both arms

Dose constraints to organs at risk are estimated considering the experience of our institution as well as the data reported by Emami et al. [17]. The dose to the eyes, temporal lobes, salivary glands, mandible has to be as low as possible. Optic nerves, chiasm and brainstem constraint is ≤ 54 Gy. The brainstem surface (1% of volume) contacting the tumour is allowed to receive >54 Gy, with Dmax ≤ 60 Gy. The doses at the brainstem center has to be <50 Gy. The spinal cord dose constraint is ≤ 45 Gy with 1% of volume allowed to receive >45 Gy (Dmax ≤ 50 Gy).

## Organization and follow-up

Patient data will be collected and documented pseudonymously using electronic data processing (e.g. patient initials, date of birth and study number) at the study office at HIT.

The study data as for example all medical reports, RT documentation and CRF's will be collected at the study office at HIT.

Local recurrences will be confirmed radiologically and histologically whenever possible. At least two medical doctors (radiation oncologist and/or radiologist) will be required to judge of the recurrence or toxicity. Each adverse event occurring in connection with the therapy has to be documented, independent of the cause.

The first and the second follow-up examination will be performed 4-6 weeks and 3 months after completion of RT (follow-up 1 and 2). Follow-up examinations will then be scheduled after 6 months (follow-up 3), 9-12 months (follow-up 4), and then once a year for further 3 years (follow-up 5, 6 and 7). Additional visits will be scheduled as necessary. Acute toxicity is assessed at least weekly during RT and documented at the end of the RT series, 6 weeks after completion of RT and 3 months after RT. Late toxicity will be documented in regular intervals of 6 or 12 months during the observation period. All the patients will be observed for radiation specific acute and late AEs for the time of at least 5 years after irradiation. The maximum grade of toxicity will be determined for each patient.

The Common Terminology Criteria for Adverse Events V4.0 (CTCAE V4.0) will be used to grade acute toxicity from radiation therapy. The criteria are relevant from the 8th irradiation day until day 90, i.e. until the 1st follow-up visit. Thereafter, the RTOG/EORTC Criteria of Late Effects will be utilized. All acute radiation effects will be documented on an Acute Radiation Effect-Form. In addition, the AE-Form and/or SAE-Form will be filled out.

RTOG/EORTC Late Morbidity Scoring Scheme will be used to grade toxicity from radiation therapy occurring later than 90 days after its start, i.e. beyond the 1st follow-up visit. All late radiation effects will be documented on a Late Radiation Effect-Form. Every patient will be followed for LPFS and AEs for a time period of 5 years. Furthermore, patients will be followed for survival and locoregional recurrences for a time period of 5 years after completion of the irradiation.

Accrual period for the trial will be approximately 7 years starting in autumn 2010.

The individual reasons for the study interruption are patient death or the withdrawal of the patient to participate in the study. Withdrawals, lost to follow-ups and patients for whom no event has occurred at study termination are treated as censored observations. The censoring date is given by the last known date at which no event has occurred for the respective patient.

With proven recurrence of disease or the development of distant metastases, the patient will be censored for our study and will be eligible for any additional appropriate therapy or inclusion in other investigative protocols, but should still be followed in order to document survival and radiation specific AEs.

High incidence of unknown AEs or increase in known AEs with the disadvantageous proportion between risk and benefits of the proposed radiation therapy or unacceptable high rates of SAEs can terminate the study earlier.

## Statistical considerations

This trial is a phase III study to demonstrate that ion radiotherapy (experimental treatment) is not relevantly inferior to proton radiotherapy (standard treatment) with respect to the 5 year LPFS rate. The null-hypothesis to be assessed in confirmatory analysis states that the 5 year LPFS rate in the experimental treatment group is at least 5% lower than the 5 year LPFS rate in the standard treatment group. Confirmatory analysis for the primary end point is based on the intention-to-treat population. A group-sequential design is applied with one interim analysis after half of the expected number of events has occurred to allow for an early stopping of the study in case of an overwhelming treatment effect. The stopping rule is according to O'Brien and Fleming [18]. The one-sided critical level in the interim analysis is given by 0.26%, and in the final analysis it is given by 2.4%. Test decision is made by comparing the corresponding repeated confidence intervals to the boundary of the non-inferiority range of -5% (LPFS rate experimental treatment - LPFS rate standard treatment). With this design, the one-sided overall type I error rate is controlled by 2.5%. The confidence intervals for the differences between the LPFS rates are calculated based on the log(-log)-transformed Kaplan-Meier estimates of the rates using Greenwood's formula to estimate the underlying variance [19].

The total sample size of 154 patients provides a power of 80% to show non-inferiority of the experimental treatment to the standard treatment if the experimental treatment is in fact superior with respect to the 5 year LPFS rate by 7% and if the LPFS rate of the standard treatment group is 90%. Calculation of the sample size is based on the non-inferiority test for the differences between rates as described by Farrington and Manning [20].

If the confirmatory aim of the study cannot be reached, the trial still allows a descriptive head-to-head comparison of the two treatments, and the inspection of the confidence interval for the treatment effect enables to exclude a certain amount of difference in efficacy between the treatments.

To assess the impact of major protocol deviations, an analogous analysis of the primary outcome variable will be performed for the per protocol set. Analysis of the secondary endpoints as overall survival, progression-free and metastasis-free survival will be performed analogously to the primary endpoint. The survival curves will be estimated using the Kaplan-Meier product-limit method [21].

All documented variables will be analyzed descriptively by tabulation of the measures of the empirical distributions according to the scale level of the variables. Descriptive p-values of the corresponding statistical tests comparing the treatment groups and associated 95% confidence intervals will be given. The homogeneity of the treatment groups will be described by comparison of the demographic data and the baseline values of the measured variables.

Safety analysis and analysis of toxicity will be based on the data set of all randomized patients who were treated with the experimental or the standard treatment at least once. The safety analysis includes calculation and comparison of frequencies and rates of adverse and serious adverse events reported in the two treatment groups.

All analyses will be done using SAS version 9.1 or higher.

## Discussion

Proton therapy is the standard treatment option in skull base chondrosarcoma patients after the tumour resection. Our experience with carbon ion therapy in patients with chondrosarcomas showed excellent results. However, until now, it has been not possible to compare both particles, i.e. protons and carbon ions, directly within the same facility for the treatment of low grade skull base chondrosarcomas.

This trial is a phase III study to demonstrate that carbon ion radiotherapy (experimental treatment) is not relevantly inferior and at least as good as proton radiotherapy (standard treatment) with respect to 5 year LPFS in the treatment of chondrosarcomas. Additionally, we expect less toxicity in the carbon ion treatment arm due to the reduced lateral scattering of the carbon beam.

## Competing interests

The authors declare that they have no competing interests.

## Authors' contributions

AVN, GR, MK, MWM, ADJ, SEC, and JD have developed the study concept. AVN, MWM, GR and JD wrote the study protocol and obtained ethics approval. GR, MK participated in the design of the study and performed the statistical analysis. AVN, ADJ, MWM, SEC and JD will provide patient care. AVN, GR, MK, MWM, ADJ, SEC and JD will implement the protocol and oversee collection of the data. All authors read and approved the final manuscript.

## Pre-publication history

The pre-publication history for this paper can be accessed here:

http://www.biomedcentral.com/1471-2407/10/606/prepub
